# Understanding *Mucor circinelloides* pathogenesis by comparative genomics and phenotypical studies

**DOI:** 10.1080/21505594.2018.1435249

**Published:** 2018-04-18

**Authors:** Loida López-Fernández, Marta Sanchis, Patricia Navarro-Rodríguez, Francisco E. Nicolás, Fátima Silva-Franco, Josep Guarro, Victoriano Garre, María Isabel Navarro-Mendoza, Carlos Pérez-Arques, Javier Capilla

**Affiliations:** a Unitat de Microbiologia, Facultat de Medicina i Ciències de la Salut, Universitat Rovira i Virgili and Institut d'Investigació Sanitària Pere Virgili (IISPV), Reus, Spain; b Departamento de Genética y Microbiología, Facultad de Biología, Universidad de Murcia, Murcia, Spain; c Centre for Genomic Research, University of Liverpool, Liverpool, UK

**Keywords:** Mucor, Mucormycosis, Virulence factor, Genome comparison, Murine model infection, Fungal pathogenesis, Virulence factors, Whole genome sequencing

## Abstract

The increasing number of infections by species of Mucorales and their high mortality constitute an important concern for public health. This study aims to decipher the genetic basis of *Mucor circinelloides* pathogenicity, which displays virulence in a strain dependent manner. Assuming that genetic differences between strains may be linked to different pathotypes, we have conducted a study to explore genes responsible for virulence in *M. circinelloides* by whole genome sequencing of the avirulent strain NRRL3631 and comparison with the virulent strain CBS277.49. This genome analysis revealed 773 truncated, discontiguous and absent genes in the NRRL3631 strain. We also examined phenotypic traits resulting in reduced heat stress tolerance, chitosan content and lower susceptibility to toxic compounds (calcofluor white and sodium dodecyl sulphate) in the virulent strain, suggesting the influence of cell wall on pathogenesis. Based on these results, we focused on studying extracellular protein-coding genes by gene deletion and further pathotype characterization of mutants in murine models of pulmonary and systemic infection. Deletion of gene ID112092, which codes for a hypothetical extracellular protein of unknown function, resulted in significant reduction of virulence.

Although pathogenesis is a multifactorial process, these findings highlight the crucial role of surface and secreted proteins in *M. circinelloides* virulence and should promote further studies of other differential genes.

## Introduction

Mucormycosis is an emerging opportunistic infection caused by fungi of the order Mucorales, which is becoming more frequent due to the increase in population with risk factors, such as immunosuppression, diabetes, blunt trauma and hematological malignancies. *Mucor circinelloides* is one of the most frequent species within Mucorales causing fatal mucormycosis. Mucorales infections are difficult to treat due to their fast dissemination within the host tissues and their low susceptibility to antifungal agents, which drives to worse outcomes than other common fungal diseases, such as aspergillosis [1-3]. Experimental mucormycosis has demonstrated differences in virulence between strains, which points to individual factors responsible for pathogenesis. This phenomenon has been previously reported by Li *et al* [4], who observed different pathogenicity among *M. circinelloides* strains in *Galleria mellonella* infections. The authors also observed phenotypical characteristics related to virulence, such as higher germination velocity, spore size and protein secretion.

In the present study we evaluated the virulence of two *M. circinelloides* f. *lusitanicus* strains by systemic and pulmonary infection murine models. One of the strains was previously identified as highly virulent (CBS277.49) and the other one as avirulent (NRRL3631) in moth wax infection [4]. Once we verified the pathogenic capacity of both strains in the murine model, we carried out the whole genome sequencing of the avirulent strain (NRRL3631) and we conducted a genome comparison study with the virulent one (already sequenced). In this analysis we aim to summarize genetic differences between both *M. circinelloides* strains, directed for screening potential determinants of pathogenicity. Nevertheless, interpreting and understanding the role of genes in virulence is a difficult challenge, even for those encoding proteins with known function. Considering the strong association between extracellular proteins, cell wall structure and pathogenic potential [5], we have addressed the study of genes coding for extracellular enzymes using gene deletion and replacement method. This approach has allowed us to examine the effect of gene lack on the virulence of the pathogenic strain. Overall, the results of the genetic screening carry out in the present study can provide a new route for understanding *M. circinelloides* pathogenesis and control of mucormycosis.

## Results

### CBS277.49 and NRRL3631 virulence in pulmonary and systemic murine models

**Figure 1.**
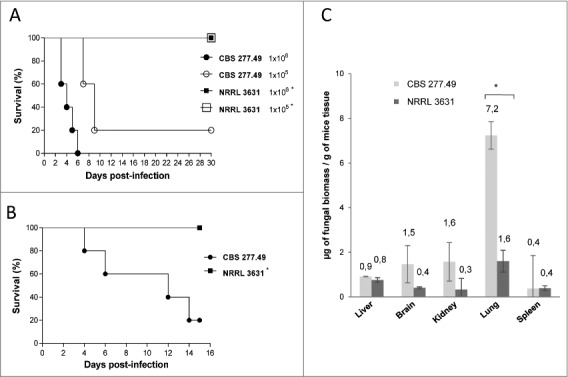
Virulence of *M. circinelloides* CBS277.49 and NRRL3631 strains in neutropenic murine host. Survival of mice infected (A) intravenously (i.v.) with 1 × 10^6^ and 1 × 10^5^ CFU of fungal strains and (B) intranasally (i.n.) with 5 × 10^7^ CFU of fungal strains show significant lower virulence of NRRL 3631 (*) (*p* < 0.05). (C) Fungal biomass quantification of liver, brain, kidney lung and spleen 3 days after i.v. infection with 1 × 10^6^ CFU/animal of CBS277.49 and NRRL3631 using real-time PCR. Data represent µg of fungal biomass respect 1 g of mice tissue biomass. Significant differences are indicated (*). Bars indicate the standard deviation from independent experiments.

Systemic and pulmonary infections with spores of CBS277.49 strain resulted on 100% mortality after challenge with 1×10^6^ CFU/animal administered intravenously (i.v.), and 80% mortality after i.v inoculation with 1 × 10^5^ CFU/animal or intranasally inoculation with 5 × 10^7^ CFU/animal. However, infections performed with strain NRRL3631 resulted in 100% survival at any inocula and route of infection assayed (Fig. 1A and 1B).

Fungal burden was quantified by real time PCR (qPCR) in relevant organs of mice infected i.v. with both wildtype strains (1 × 10^5^ CFU / animal). Quantification of total genomic DNA (gDNA) and conversion to biomass values revealed greater amount of CBS277.49 in lung (7.2 µg of fungal biomass / g of mice tissue) in comparison to NRRL3631 strain (0.5 µg biomass / g of mice tissue) (p = 0.019) (Fig. 1C). Lower amount of fungal biomass was detected in kidney, brain, liver and spleen with no significant differences among strains (*p* ≥ 0.33). Taken together, these mortality rate and fungal load results indicate higher infection capacity of CBS 277.49 in mice, being lung the preferred target organ.

### Small and large spores from CBS277.49 strain display different germination kinetics but same infectivity.

**Figure 2.**
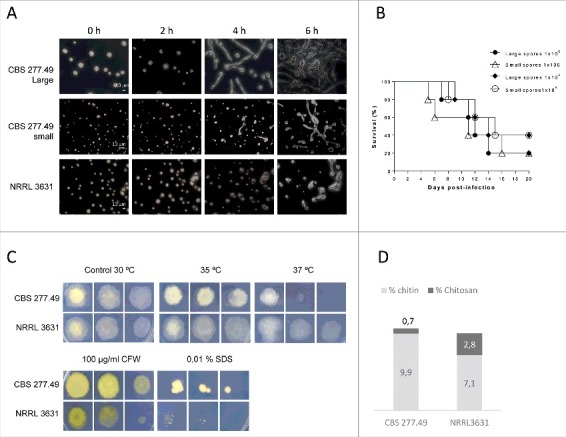
Fungal strains phenotypes. Small and large spores of CBS277.49 strain exhibit differences in (A) germination kinetics and (B) similar virulence capacity in murine model infection. (C) Fungal colonies from CBS277.49 and NRRL3631 *M. circinelloides* strains grown for 2–3 days at 30°C on SM plates containing sodium dodecyl sulphate (SDS) or calcofluor white (CFW) and 5 days at 35ºC and 37ºC, inoculating 10^3^, 10^2^ and 10 fresh spores. (D) Percentage of chitin and chitosan respect to initial mycelial dry weight.

Microscopic observation revealed different germination rate between small (5 × 3.5 µm) and large (12.3 × 9 µm) sporangiospores from the pathogenic strain (CBS277.49). Large sporangiospores from CBS277.49 produced visible germ tubes after 3–4 h of incubation, while small ones showed a 2 hours delay (Fig. 2A). The NRRL3631 strain, which only produces one-sized sporangiospores (5 × 3.5 µm), showed similar germination kinetics to CBS277.49 small sporangiospores i.e., around 6 hours post-incubation. Despite these differences, the systemic infection by small or large sporangiospores of the pathogenic strain resulted in similar virulence at both inocula (1 × 10^5^ and 1 × 10^6^ CFU/animal) (*p* ≥ 0.602) (Fig. 2B). These results showed no correlation between sporangiospore size and pathogenic capacity, suggesting the existence of virulent factors inherent to CBS277.49 strain not related to spore dimensions and germination kinetics.

### Whole genome comparison between CBS277.49 and NRRL3631

**Figure 3.**
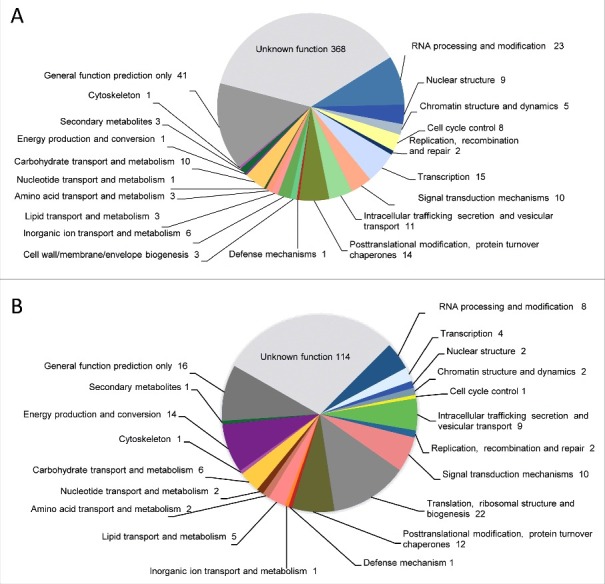
Genes identified in the genome comparison study clustered in functional categories based on KOG classification. (A) Absent genes and (B) discontiguous genes in NRRL3631 strain.

Whole-genome resequencing of *M*. *circinelloides* NRRL3631 was performed by shotgun paired-end method using Illumina sequencing technology. The 91% of the reads were aligned to the reference genome, covering the 96% of the genome size (36.6 Gb) (Table 1). This search lead to the detection of single nucleotide polymorphisms (SNPs) and unmapped regions, including four scaffolds entirely unmapped of CBS277.49 (scaffolds 17, 19, 21 and 22) containing 17,493 Kb, 9,869 Kb, 7,638 Kb and 4,155 Kb, respectively (Suppl. Fig. S1). The uncovered gaps were examined and 543 genes were identified as unique genes in the virulent strain that were classified in functional categories based on KOG (Fig. 3A). Among them, we identified a high proportion of protein coding genes with unknown functions that accounted for the 68% of total number of unique genes in CBS277.49. The genes that were exclusively found in the pathogenic strain with known functions are listed in Table 2. These CBS277.49-specific genes code for proteins involved in RNA processing, nuclear structure and chromatin dynamics, cell cycle control, signal-transduction pathways, post-translational modifications enzymes, vesicle formation and secretory pathway sorting, transport and metabolism, including enzymes involved in extracellular structures biogenesis, secondary metabolites biosynthesis and defense against oxidant compounds (Table 2).

**Table 1.** Assembly and mapping statistics of *M. circinelloides* NRRL3631 genome sequencing.

Assembly Features	
Nº scaffolds	3,429
Longest scaffold	61,306
Shortest scaffold	300
Number of scaffold > 1 Kb	89.7 %
Number of scaffolds > 10 Kb	38.8 %
N50	18,177
L50	1,6481,489 bp
Mapping features	
Total reads	56,409,304
Mapped reads	51,384,356
Mapped reads %	91
Mean depth	134
Total gaps size	1,530,826
Nº of gaps > 50 bp	2,751
Homozygous	176,188
Heterozygous	24,694
Indels	16,086

**Table 2.** Eukaryotic Orthologous Groups (KOG) classification of absent or discontiguos genes detected in NRRL3631 genome in comparison to CBS277.49. A total of 395 sequences assigned to KOG classifications within the principal 17 categories are shown.

KOG	(no.) Absent genes	(no.) Discontigous genes
RNA processing	(2) ATP-dependent RNA helicase	(2) RNA adenine N-6-methyltransferase
	(1) RRM motif-containing protein	(2) Splicing factor
	(1) Alternative splicing factor SRp20/9G8	(1) Polyadenylate-binding protein (RRM)
Nuclear Structure	(7) Nucleolar GTPase/ATPase p130	(2) Nucleolar GTPase
	(2) Nuclear pore complex p54 component	
Chromatin structure and dynamics	(3) DNA-binding centromere protein B	(1) SNF transcription factor
	(2) Chromatin-associated protein Dek	(2) Histone 2A
Cell cycle control and cell division	(3) DNA helicase PIF1/RRM3	(1) ATP-dependent DNA helicase
	(1) Serine/threonine protein kinase Chk2	(2) Transcription initiation factor IIF subunit
	(1) Chromosome condensation complex	(1) Transcription factor Myb superfamily
	(1) P53-interacting protein 53BP/ASPP	(1) Nuclear receptor coregulatory SMRT/SMRTER
	(1) Proline-serine-threonine phosphatase interacting protein	(1) Proline-serine-threonine phosphatase interacting protein
	(1) Caspase C14	
Replication, repair and recombination	(1) Ribonuclease H	
	(1) Mismatch repair ATPase MSH6	
Transcription	(2) Nuclear localization sequence binding protein	(1) Transcription initiation factor TFIID
	(1) Chromodomain-helicase DNA-binding protein	(1) Transcription factor, Myb superfamily
	(3) Predicted forkhead transcription factor	(1) Nuclear receptor coregulator SMRT/SMRTE
	(1) Histone acetyltransferase	
	(2) Heat shock transcription factor	
	(4) Transcription factor	
	(1) Nuclear receptor coregulator	
Signal transduction mechanisms	(3) Serine/threonine protein kinase	(1) G-protein
	(2) Ca^2+^ bindig protein	(1) Lipoprotein receptors
	(1) C-type lectin	(1) cAMP-dependent protein kinase (PKA)
	(2) GTP-binding protein	(1) Calmodulin
	(1) FOG: Hormone receptors	(2) C-type lectin
	(1) Glycosylphosphatidylinositol anchor synthesis protein	(2) Guanine nucleotide exchange factor
		(1) Serine/threonine protein phosphatase 2A
Intracellular trafficking secretion and vesicular transport	(1) Rab3 effector RIM1	(1) Vesicle coat complex COPI
	(2) GTPase Rab5	(1) Clathrin
	(1) Vesicle coat complex AP-3	(2) GTPase Rab6
	(1) Vesicle coat complex COPI,	(1) Peroxin
	(1) Vacuolar assembly/sorting protein VPS9	(1) Mitochondrial import inner membrane translocase
	(2) Clathrin coat binding protein	
	(2) Guanine nucleotide exchange factor	
Translation, ribosomal structure and biogenesis		(13) Ribosomal protein
		(5) Elongation factor
Post-translational modification, protein turnover chaperones	(8) E3 ubiquitin ligase	(3) Chaperone
	(2) Chaperones HSP70/HSC70	(1) Ubiquitin-protein ligase
	(1) Beta-1, 6-N-acetylglucosaminyltransferase	(1) Peptidyl-prolyl cis-trans isomerase
	(1) Protein farnesyltransferase	(1) Serine palmitoyltransferase
		(1) Prohibitin
		(1) Myosin phosphatase
		(2) AAA ATPase
Defense mechanisms	(1) Pyrazinamidase/nicotinamidase PNC1	(1) Von Willebrand factor
Extracellular structures and cell wall biogenesis	(2) Collagen (type IV and type XIII)	
	(1) Phospholipid scramblase	
Energy production and conversion	(1) Voltage-gated shaker-like K^+^ channel	
Secondary metabolites biosynthesis, transport and catabolism	(2) Alpha-aminoadipate reductase	(1) Beta-carotene 15'-dioxygenase
	(1) Flavin-containing monooxygenase	
Cytoskeleton	(1) Projectin	(1) Rho GTPase effector BNI1
Transport and metabolism	(4) Ion channels	(2) Lipase
	(5) Permeases	(1) 3-oxoacyl CoA thiolase
	(1) Monocarboxylate transporter	(2) Gamma-glutamyltransferase
	(1) Protein kinase	(1) dUTPase
	(2) Acyl-CoA-binding protein	(1) Nucleoside transporter
	(1) 3-oxoacyl CoA thiolase	(1) Permease
	(3) Glutamate synthase	(1) Monocarboxylate transporter
	(2) β*-*1, 6-N-acetylglucosaminyltransferase	(2) β-1, 6-N-acetylglucosaminyltransferase
	(2) Chitinase	(1) Cytochrome c oxidase
	1) Glycoside hydrolase/ deacetylase	(2) Dihydrolipoamide acetyltransferase
		(2) Mitochondrial carrier
		(2) Dihydrolipoamide acetyltransferase
		(1) NADH dehydrogenase
		(7) ATP synthase
		(4) F0F1-type ATP synthase, beta subunit
		(1) NADH-ubiquinone/plastoquinone oxidoreductase
Other functions	(10) Reverse transcriptase	(1) Phospholipase D1
	(14) Transposon-encoded protein	(3) Reverse transcriptase
	(2) Exonuclease	(4) Transposon-encoded protein
	(17) Zn-finger protein	(7) Zn-finger protein
	(1) UV radiation resistance associated protein	(1) Monodehydroascorbate/ferredoxin reductase
	(10) Endonuclease	(1) Transferrin receptor
	(1) Phospholipase D	(1) Thiopurine S-methyltransferase activity
	(1) Carbamoyl phosphate synthetase	
	(1) Hypothetical transmembrane drug transporter	
	(1) Hypothetical transcriptional activator of glycolytic enzymes	

To further analyze variation between both genomes, we conducted a pairwise comparison between NRRL3631 reads assembled into contigs and the reference genome. This analysis allows highlighting the variation in gene position, resulting in 230 discontiguous protein-coding sequences that were clustered by KOG classification (Fig. 3B). Approximately the 50% of these discontiguous genes code for proteins with unknown function. In the functionally annotated genes we found many translation and ribosomal proteins, followed by post-translational modification, protein turnover, chaperones as well as energy production and conversion enzymes (Table 2). The total extracellular enzymes found in the analysis are listed in Table 3.

**Table 3.** Genes coding for extracellular enzymes identified in the genome comparison study among CBS277.49 and NRRL3631 genomes as absent, or discontiguous sequences in NRRL3631. ^a^Number of duplicated genes in CBS277.49 *M. circinelloides* genome based upon BLAST with E-value < E^−50^ and ≥ 70% identity. ^b^Distribution of orthologues within the fungi species based upon BLAST with E-value < E^−22^ and ≥ 50% identity. Ac: Absidia glauca, Cc: *Choanephora cucurbitarum*, Cg: *Colletotrichum gloeosporioides*, Lc: *Lichtheimia corymbifera* Lr*: Lichtheimia ramosa*, Ma*: Mucor ambiguos*, Mc: *Mucor circinelloides*, Me: *Morteriella elongata*, Pb: *Phycomyces blaskespeanus*, Pp: *Parasitella parasítica*, Rm: *Rhizopus microsporus*, Ri: *Rhizopus irregularis*, Rd: *Rhizopus delemar*, Ro; *Rhizopus oryzae*, Um: *Ustilago maydis*.

Protein ID	KOG ID	Protein function	No. of Paralogues^a^	Fungal taxonomic distribution of best hits^b^
Unique genes in CBSS277.49				
167922	KOG2126	Glycosylphosphatidylinositol anchor synthesis protein	—	Mc, Ma, Pp, Cc, Rm
167058	KOG1216	von Willebrand factor, related coagulation proteins	—	Mc, Pp, Cc, Ma, Rd, Rm, Lc
115405	KOG3599	Ca^2+^ cation channel polycystin, Mucine	—	Mc
112425	KOG1218	Proteins containing Ca^2+^-binding EGF-like domains	—	Mc, Ma, Pp
76897	KOG2806	Chitinase	—	Mc
185052	—	Putative glycoside hydrolase/deacetylase,	2	Mc, Ma, Pp, Rm, Pb
163412	—	Chitin binding protein, putative peritrophin-A	2	Mc
112072	—	Chitin binding protein, putative peritrophin-A	1	Mc, Ma
113101	—	Chitin binding protein, putative peritrophin-A	1	Mc
110615	—	Putative MFS general substrate transporter	1	Mc, Ma, Pp, Cc
80244	—	Putative membrane transporter	—	Mc, Pp
189787	—	Unknown	—	Mc, Cc, Lc, Pb
155646	—	Unknown	2	Mc, Ma Pp, Rd Cc, Ac, Pb, Lr, Lc
104593	—	Unknown	—	Mc
83937	—	Unknown	—	Mc
79369	—	Unknown	—	Mc
77057	—	Unknown	2	Mc, Ma, Rd, Rm, Fo, Af
38654	—	Unknown	1	Mc, Ma, Cc, Lc, Pp, Ac, Rm, Rd
Discontiguos genes in NRRL3631				
155853	KOG4157	β-1, 6-*N*-acetylglucosaminyltransferase	—	Mc, Pp, Rd, Cc, Rm
155032	KOG3083	Prohibitin	1	Mc, Pp, Rd, Ma, Rm, Cc, Ag, Pb, Me, Lc, Pp Pb, Ri, Cg, Um
141273	KOG1285	β-carotene 15-dioxygenase	—	Mc, Ma, Pp, Rd, Rm, Ro, Rm, Ag
108920	KOG2410	hypothetical γ-glutamyltransferase	2	Mc, Pp, Rm,Ma, Pb, Ag, Cc, Rd
83381	KOG1550	Extracellular protein SEL-1	—	Mc, Pp, Cc, Rm, Pb, Ag, Lr
39631	KOG3339	Predicted glycosyltransferase Alg14	—	Mc, Pp, Cc, Pb, Lc, Ag, Ri
106371	—	Unknown	2	Mc, Ma, Pp, Pb, Lr, Rm, Lc
112092	—	Unknown	—	Mc, Pp, Ag
116342	—	Unknown	—	Mc, Ma, Pp
166851	—	Unknown	4	Mc, Ma, Rm, Rd

Reads from NRRL3631 sequencing that did not map to the reference genome (2,183 reads and contigs) were aligned to NCBI's nucleotide database (nr/nt) using BLAST. The returned best hits showed significant similarity to mitochondrial proteins as cytochrome c oxidase, NADH dehydrogenase from other strains and species of Mucorales (*M. circinelloides, Rhizopus oryzae* or *Rhizopus delemar*), and some bacteria (data not shown).

### Heat stress and cell wall-related phenotypes

As shown in Fig. 2C, both strains were able to grow under heat stress conditions (35ºC and 37ºC), although the pathogenic strain exhibited slightly lower growth than NRLL3631 at both temperatures, being more evident the difference at 37°C.

Growth in presence of noxious compounds revealed higher sensitivity of NRRL3631 to SDS and CFW, which exhibited reduced colony size in comparison to CBS277.49 (Fig. 2C).

Fungal cell wall extraction and quantification revealed that both strains contained similar amount of total *N*-acetylglucosamine polymers (chitin and chitosan), which represent approximately 10% of dry weight mycelial biomass. However, the chitosan fraction was lower in CBS277.49 (0.7%) than in NRRL3631 (2.5%) (Fig. 2D).

### Sequence analysis of ID112092 and ID108920 proteins of *Mucor circinelloides*

We focused the study on extracellular proteins as virulence factor candidates due to their potential effector [6] or immunomodulating role on fungal pathogenesis [7]. The screening was based on the following criteria: surface-exposed proteins, considering both cell wall anchored proteins and secreted peptides since many extracellular enzymes remain embedded in cell wall without predicted anchor domains; gene products with unknown function; genes with less than two paralogous and poorly conserved sequences in closer fungal species [8-10]. From this first screening listed in Table 3, we next examined the deduced amino acid sequences of selected genes, focusing the study on ID112092 and ID108920. The deduced amino acid sequences of ID112092 and ID108920 were used to perform sequence and domain motif analysis. The results showed the presence of N-terminal signal peptide and several *N*- and *O*-glycosylation predicted sites along the sequences, which suggests that both genes code for highly glycosylated-secreted proteins (Suppl. Fig. S2 and S3). BLAST search against NCBI database showed that gene ID112092 is poorly conserved in other organisms being the closest match *Mucor ambiguous* (80.5% identity), followed by *Absidia glauca* (58.4% and 24.7% identity) and *Parasitella parasitica* (45.8% identity). All proteins, except the short protein SAM09789.1 from *A. glauca*, have a conserved Friend of Prmt1 motif (FoP), which corresponds to a novel chromatin target of protein arginine methyltransferase, involved in gene regulation and methylation. The study of the two orthologues genes identified in *A. glauca* genome: SAM09789.1 (hypothetical small-secreted protein) and SAL94833.1 (hypothetical intracellular protein with Fop domain) (http://fungi.ensembl.org/Absidia_glauca/Info/Index) is particularly interesting. Both genes are located in different scaffolds and display partial sequence similarity to N-terminal and C-terminal region of ID112092, respectively (Suppl. Fig. S2). These findings indicate that ID112092 is a unique gene, only present in *M. circinelloides* and *M. ambiguous* species, which contains an unusual combination of chromatin-targeting domain and a signal peptide for its extracellular secretion.

Unlike the previous gene, ID108920 is highly conserved from fungal to mammalian genomes and has a putative gamma-glutamyl transpeptidase activity. Sequence analysis revealed that all orthologues genes contain a transmembrane domain that compromises between 17–22 aminoacids, with the exception of *M. circinelloides* and *M. ambiguous* that show a signal peptide instead (Suppl. Fig. S3).

### Target deletion of genes ID112092 and ID108920 encoding extracellular proteins

**Figure 4.**
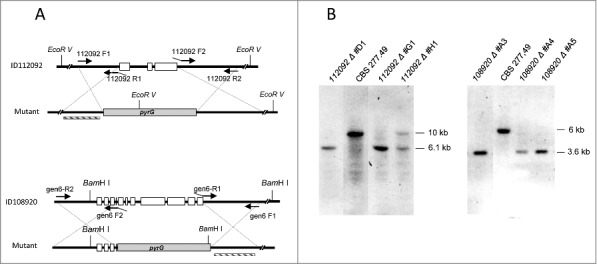
Disruption of 112092 and 108920 genes. (A) Targeted replacement strategy using a vector generated by fusion PCR with the pyrG gene as selective marker. Black arrowheads indicate the primer pairs used for amplification of DNA fragments. Probes are indicated (dashed bar). (B) Southern analysis of gDNAs from *M. circinelloides* CBS277.49 and transformants. DNAs were digested with *Eco*R V and *Sma* I to detect 112092 and 108920 gene deletions, respectively.

Target deletion of ID112092 and ID108920 was performed by replacement of the entire coding sequences by deletion vectors in MU402 strain (Fig. 4A). Initial transformants were grown in selective medium and homologous recombination was confirmed by *Southern blot* analysis using appropriate restriction enzymes and probes. The wild type strain showed a 10 Kb *Eco*RV hybridizing band corresponding to ID112092 that was replaced by a 6.1 Kb band in the homologous integrative transformants #D1 and #G1, indicating deletion of the gene ID112092. Similarly, the 6 Kb *Bam*HI fragment of wild type corresponding to the gene ID108920 was replaced by a 3.6 Kb band in the transformants #A3 and #A5, indicating homologous insertion of the disruption vector and therefore the deletion of ID108920 (Fig. 4B).

### Virulence of knockout mutants in genes coding for predicted extracellular proteins ID112092 and ID108920

**Figure 5.**
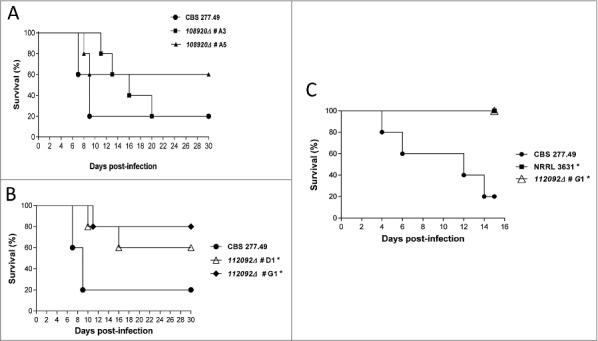
Virulence of knockout mutants in neutropenic mice infection. (A) and (B) Survival of mice infected intravenously (i.v.) with 1 × 10^5^ CFU and (C) mice infected intranasally with 5 × 10^7^ CFU of fungal strains. Data showed attenuated virulence of mutants in ID112092 and ID108920 genes. Significant differences are indicated (*).

Virulence of knockout mutants was evaluated by experimental disseminated and pulmonary infections in mice. The null mutants in the gene ID108920 (hypothetical γ-glutamyltranspeptidase) (transformants #A3 and #A5) exhibited similar virulence to the wild-type strain (CBS277.49) in disseminated infections (*p* ≥ 0.081) (Fig. 5A). In contrast, infection with spores of knockout mutants in the gene ID112092 (unknown function) (transformants #D1 and #G1) resulted in a significant reduction of mice mortality (*p* ≤ 0.043) (Fig. 5B). Pulmonary infections performed with *112092∆* mutant (transformant #G1) resulted in high reduction of virulence with 100% survival in comparison to wild type strain (p = 0.017) (Fig. 5C).

## Discussion

Only some members of *Mucorales* are able to cause disease in warm-blooded animals, including humans. These pathogen lineages have evolved by adaption to their environmental niches and acquisition of novel strategies, allowing them to cross-kingdom host jumps [11]. However, some species of the Mucorales exhibit a variety of pathogenic potential traits in different strains, suggesting that small genetic changes can be responsible for pathogenicity. In fact, a previous study correlates virulence with spore size and germination velocity of *M. circinelloides* in *G. mellonella* infection, being the strains with faster germination and larger spores more pathogenic [4]. This phenomenon has also been observed in other fungal species like *Paracoccidioides brasiliensis* and *Cryptococcus neoformans* [12, 13]. However, the link between virulence and spore size is not so straightforward in *M. circinelloides*. In fact, our results point out that virulence of CBS277.49 is independent of sporangiospores size in both systemic and pulmonary infections. In addition, we must consider that NRRL3631 remains avirulent after pulmonary infection, despite small sporangiospores reach more easily alveoli and in consequence should favor infection [14]; while the large CBS277.49 sporangiospores are highly pathogenic. The thermotolerance assay showed similar ability to grow at 35ºC and 37ºC in both strains, which is crucial to infect warm-blooded hosts. In addition, no alteration in growth capacity in nutritive or minimal media culture was observed in the avirulent strain, beside its delay in germination. Taking all together, our results suggest that other factors, not related to spore morphology or growth capacity, may be involved in the pathogenesis of *M. circinelloides*.

In an attempt to decipher novel virulence determinants responsible for the pathogenicity of the strain CBS277.49, the genomes comparison has provided strain-specific and discontiguous genes that merit to be further explored, with the aim to identify candidate virulence factors. We hypothesize that this genetic variation among strains may be the result of gene loss, as the same occurs in the obligate biotrophic human pathogen *Pneumocystis jirovecii* [15], or horizontal gene transfer, as it happens in many other fungal lineages [16, 17]. Furthermore, loss of genes can also be responsible of virulence acquisition, even though this hypothesis was discarded in *M. circinelloides* as we only detected NRRL3631 unique genes coding for mitochondrial enzymes.

Due to the overwhelming number of genetic differences found among strains, mainly related to RNA and DNA machinery, cell division, metabolism, secretion and transport, the big challenge lies on unravelling those genetic determinants involved in virulence. Taking advantage that phenotypic observations on *M. circinelloides* strains give evidence that thermotolerance, germination velocity and growth capacity do not correspond to virulence-associated traits, we discarded genes related to those phenotypes. On the other hand, cell wall differences among virulent and avirulent strain were considered relevant, since NRRL3631 displays higher sensitivity to CFW and SDS, toxic agents that bind to chitin and interfere with the phospholipid bilayer, respectively [18]. Whereby we assume that cell wall permeability and structure might be altered in the pathogenic strain. Controversially, we detected less chitosan content in the cell wall of the virulent strain, a deacetylated form of chitin that has been extensively associated to virulence in other fungal pathogens, like *C. neoformans* [19]. Recently, chitin and chitosan have been shown to exhibit different immune activation properties. Chitin seems to stimulate and activate Th2 immune response driving to less efficient protection against fungal infection, as observed O'Dea *et al* [20]. in *Aspergillus fumigatus* strains with different chitin content. Whereas chitosan, but not chitin, has been shown to induce a strong IL-β response that contributes to the activation of inflammation, generally considered protective in fungal pathogens [21, 22]. Considering these chitin and chitosan immune modulatory properties, it is possible that higher chitin and lower chitosan content on CBS277.49 cell wall are linked to its high virulence. Another hypothetical explanation to link lower amount of chitosan and virulence is that deacetylation of chitin to chitosan protects the fungal cell wall from being hydrolyzed by host chitinases, and consequently might contribute to lower levels of host innate immune response, as it occurs in plant infections [23]. However, none of the genes identified in this genome analysis seem to be directly related to the lower chitosan content in the virulent strain.

Being evident the cell wall alterations in CBS277.49 strain, and the lack of physiological basis to attribute its virulence to any growth property, we focused the screening of new virulence factors on extracellular proteins with unknown function or unknown biological role. Extracellular proteins have been crucial in Mucorales evolution, diversification and virulence [24, 25]. In fact, many of these extracellular glycoproteins trigger inflammatory reactions, acting as pattern recognition receptors and affecting disease development [26, 27]. Accordingly, extracellular enzymes uniquely present in the genome of the pathogenic strain or translocated to a different position in the avirulent one were considered the main candidates for virulence factors.

From this study, it is of particular interest the gene ID112092, found in a different locus in the NRRL3631 genome, with no paralogous and poorly conserved in other species. Curiously, this gene with unknown function contains a signal peptide and a chromatin associated FoP domain, which results controversial for a glycosylated-secreted protein. It displays close homology to *A. glauca* and *P. parasitica* sequences, both symbiotic Mucorales with reported horizontal gene transfer events [28]. Specifically, *A. glauca* contains two different proteins and each one displays exclusive homology to N-terminal and to C-terminal regions of *M. circinelloides* sequence, respectively. In basis of these findings, we hypothesize that ID112092 may be a novel multidomain protein resulted from the evolutionary combination of two ancestral proteins. Whereas the functional features of ID112092 remain unknown, the knockout mutant displayed a drastic reduction of virulence in mice infection. Nevertheless, no phenotypical differences were observed in *112092*∆ mutants in spore size or growth capacity in presence of toxic compounds (CFW, SDS) and under heat stress at 35–37ºC, comparing to the wild type strain CBS277.49 (data not shown).

In summary, our study gives evidence of different pathogenic potential of two wild type strains of *M. circinelloides* that display genetic differences, and provides candidate virulence factors related to cell wall surface and secreted proteins. These findings might be of general interest on the perspective of understanding pathogenicity of *Mucorales*, and open a new path to find novel target proteins and future attempts to develop efficient therapeutic strategies. Future studies on ID112092 gene are needed in order to understand its enzymatic activity and biological role. The functional characterization of this unique gene should provide greater detail of virulence properties of *M. circinelloides* in the context of pathogen-host interaction.

## Material and methods

### Strains and culture conditions

The wild type strains CBS277.49 and NRRL3631 of *M. circinelloides* f. *lusitanicus* were used in this study. Additionally, the leucine and uracil auxotroph MU402, derived from CBS277.49, was used as recipient strain for transforming experiments [29].

To obtain fungal biomass, a sporangiospores suspension was poured into potato dextrose broth (PDB) and incubated at 30ºC under agitation at 150 rpm for 48 h. To obtain sporangiospores, strains were grown in yeast extract peptone glycerol agar (YPG) or potato dextrose agar (PDA, Pronadisa) at 30ºC for 5–7 days. Extraction of gDNA, was performed from cultures grown in PDB at 30ºC as previously described [29]. For transformation experiments *M. circinelloides* was grown on MMC medium (1% casamino acids, 0.05% yeast nitrogen base without aminoacids and ammonium sulphate, 2% glucose) [30].

### Animal models

Four week-old male OF1 mice weighing 30 g (Charles River, Criffa S.A) were used. For the systemic infection mice were immunosuppressed 2 days prior the infection by intraperitoneal injection of 200 mg/kg body weight of cyclophosphamide (Genoxal®; Laboratories Funk S.A.) and once every 5 days thereafter. Mice were challenged intravenously (i.v.) via the lateral tail vein with 1 × 10^5^ or 1 × 10^6^ sporangiospores/animal in 0.2 mL of sterile saline.

Due to the heterogeneous size of sporangiospores from strain CBS277.49, in an additional experimental assay mice were infected i.v. with previously separated small and large sporangiospores both at 1 × 10^5^ and 1 × 10^6^ CFU/animal. Sporangiospores were fractioned by filtration through two Monodur filters (10 µm pore diameter) and consecutive centrifugations at 340*g* on two size range i.e., small (5 × 3.5 µm) and large (12.3 × 9 µm) sporangiospores.

For pulmonary infections mice were immunosuppressed by subcutaneous injection of 125 mg/kg of cortisone acetate 4 days prior to the infection and once every 2 days thereafter [31]. Infection was performed in mice anesthetized with sevoflurane (Sevorane, Abbott lab.) by instillation of 0.03 mL of sterile saline containing 5 × 10^7^ sporangiospores. All experimental groups of animals were checked twice daily for 20–30 days post-infection.

The murine models of systemic and pulmonary infection were used to evaluate the virulence of knockout mutants in comparison to wild type CBS277.49 and NRRL3631 strains.

Mice care procedures and experimental conditions were supervised and approved by the Universitat Rovira i Virgili Animal Welfare and Ethics Committee. Experiments were repeated three times with similar results. Data presented are from one representative experiment.

### Fungal burden quantification

Mice infected i.v. with 1 × 10^5^ and 1 × 10^6^ sporangiospores of both wild type strains were euthanatized 6 and 3 days post-infection respectively by CO_2_ anoxia. Spleen, lungs, liver, brain and kidneys were aseptically removed and weighed for fungal burden quantification by qPCR. Organs were ground up on liquid nitrogen and gDNA was extracted as previously described [32]. Specific primers of *M. circinelloides* chitin synthase gene (ID153118) and of mice β2-microglobulin gene (ID12010) were used (Table 4). Samples analysis were carried out in triplicate in 15 µl PCR reactions containing 180 ng of test sample gDNA using SybrGreen kit (Fast SYBR® Green Master Mix -ABI) in a StepOne™ Real-Time PCR System (ABI). gDNA from non-infected mice was used as negative control. Relative amount of fungal and mice gDNA was quantified on the basis of their standard curves, elaborated with known fungal DNA concentrations (0.01 ng – 10 ng) and mice (1 ng – 200 ng) and their corresponding amplification cycle threshold (Ct). The amount of gDNA calculated for each test sample was translated into mg of biomass using the DNA: biomass ratio estimated for each organism (127 µg gDNA/ 1mg fungal biomass and 100 µg gDNA/ 1 mg mice tissue).

**Table 4.** Oligonucleotides used in this study. Italics and lower case indicate nucleotide sequences added for fusion purposes. Positions are referred to the start codon, (+) downstream or (-) upstream of ATG. Orientation is indicated, (s) sense, (as) antisense.

Name	Sequence (5′→3′)	Position from ATG	Experimental use
ChsV-1	CAAGGACGAAAAGAGAGTAAC	+ 2769 (s)	RT-PCR
ChsV-3	TGTTGGTAGTTGTGATAATCGT	+ 2985 (as)	RT-PCR
B2m-F	TTTTCATCTGTCTTCCCCTGT	+ 3105 (s)	RT-PCR
B2m-R	GTATGTATCAGTCTCAGTGGG	+ 3362 (as)	RT-PCR
pyrG-F2	TGCCTCAGCATTGGTACTTG	- 670 (s)	Deletion vector
pyrG-R2	GTACACTGGCCATGCTATCG	+ 1335 (as)	Deletion vector
112092-F1	TGAGTTCTGCTTCTTCGTCTG	- 970 (s)	Deletion vector and probe
112092-R1	*caagtaccaatgctgaggca*GGGGCTTTCTGGATGATGAGT	- 19 (as)	Deletion vector and probe
112092-F2	*cgatagcatggccagtgtac*GTAGTAGCGTTGAATAAAGAGT	+ 1327 (s)	Deletion vector
112092-R2	GTGAGTTTCTGCTTGAATTTGT	+ 2456 (as)	Deletion vector
112092-F3n	ACGAAGAATCAGCAATTACACA	- 917 (s)	Deletion vector
112092-R3n	ACTCCATCAACTTTTATAAGCC	+ 2398 (as)	Deletion vector
108920-F1	TTTTGCAGGCTTTTATTACATTT	- 1063 (s)	Deletion vector
108920-R2	ACCTATGCAATGCCTCCGATGCGA	- 791 (s)	Deletion vector
108920-F2	*cgatagcatggccagtgtac*CCATTGGCATCACGGATCAGCA	+ 563 (as)	Deletion vector
108920-R1	*caagtaccaatgctgaggca*TGCTGCTGCGTACTGACTGATGT	+ 2312 (s)	Deletion vector and probe
108920-F1	GATGCAGGTTGTGATGGAAC	+ 3123 (as)	Deletion vector and probe

We determined the average number of nuclei per hyphae by DAPI staining [33] and microscopic observation. The differences among strains were negligible, displaying similar number of nuclei (20 nuclei/ 100 µm) and amount of gDNA (127 µg/g) (Suppl. Fig. S4 and Suppl. Table S1). Experiments were repeated three times with similar results.

### Whole-genome sequencing, assembly and comparative genomics

The whole genome of the strain NRRL3631 was sequenced using Illumina technology platform (High seq) (Macrogen, Korea). A Truseq DNA library with 200 bp insert fragments was constructed and Paired-End sequenced producing 56 million sequencing reads with a read length of 100 bp. Adaptors were removed with Trimmomatic [34] and quality checked with FastqC [35]. De novo assembly was performed using SOAPdenovo2 software [36] (kmer size of 49). Reads with quality less than 30 over 70% of the read were filtered using NGS QC Toolkit [37]. Final assembly was generated using ABACAS and IMAGE (PAGIT) [38], using *Mucor circinelloides* CBS277.49 (GenBank assembly accession GCA_001638945.1) as reference. ABACAS was run twice, with an intermediate IMAGE step to close additional gaps. For running IMAGE, contigs of less than 500 bp were removed, kmer parameter was set to 51 and reads with quality less than 30 over 70% of the read were filtered using NGS QC Toolkit [37]. ABACAS was run with nucmer and sensitive settings. Whole-genome sequencing reads from the strain NRRL3631 were aligned to the reference genome (http://genome.jgi.doe.gov/vista_embed/?organism = Mucci2) using Burrow-Wheeler Aligner (BWA) software, version 0.7.13 [39] using bwa mem with the default parameters and the alignment results were visualized using the program Tablet [40]. Regions with no read aligned were examined and considered unique regions in the CBS277.49 genome. Variant calling to identify single nucleotide polymorphisms (SNPs) were conducted using Samtools 1.3 calling method, using mpileup function with default parameters [41]. Unmapped reads were assembled and analyzed by Blast search against NCBI database and sequences with best hits E ≤ e-48 were identified. Pairwise genome comparison with ACT, Artemis Comparison Tool [42], was performed aligning the NRRL3631 contigs against the reference genome (CBS277.49).

### Germination and sensitivity assays

For the germination kinetics study, 10 µl containing 5 × 10^4^ spores were incubated at 30ºC on 1% agarose and 0.5% casamino acids media (Difco Laboratories). Spore growth was monitored by optical microscopy every hour over a period of 6 hours. Due to the variable size of spores produced by CBS277.49, growth kinetics was determined separately from small (5 × 3 µm) and large sporangiospores (13 × 9 µm) and from sporangiospores of NRRL3631, which show homogeneous size (5 × 3 µm), following the procedure for sporangiospores fractioning indicated in animal model section.

For cell wall-associated sensitivity assays, 10 µl containing 10^3^, 10^2^ and 10 fresh sporangiospores were spotted onto plates of Arne synthetic medium (SM) supplemented with either 100 µg/mL calcofluor white (CFW) or 0.01% sodium dodecyl sulphate (SDS), and incubated at 30°C for 2 days. For heat stress assay, SM plates inoculated with the same procedure previously described were incubated at 35°C and 37ºC for 5 days.

### Cell wall fractioning and chitin and chitosan purification

For chitin and chitosan quantification, mycelia from both wild-type strains of *M. circinelloides* were grown in PDB for 24 h, filtrated, washed with distilled water and freeze-dried. The dry mycelia (3 g) was grounded on a IKA A11 grinder and treated with 0.5 M NaOH at 90ºC with shaking during 2 h, as previously described [43]. The cell walls fraction obtained was collected by 10.000*g* centrifugation and dialyzed against distilled water (14 kDa cut-off). Subsequently, the water insoluble fraction was collected by 10.000*g* centrifugation and subjected to chitosan-chitin extraction by 1M HCl treatment following the previously described protocol [44]. The resulting soluble (chitosan) and insoluble (chitin) fractions in HCl were collected by 10.000*g* centrifugation, dialyzed against distilled water (14 kDa cut-off), freeze-dried and weighted.

### Nuclei acid manipulation and cloning

Total gDNA was extracted from *M. circinelloides* mycelium according to previously reported protocol [45]. The DNA quality and quantity was determined by running aliquots in RedSafe-stained agarose gels and by spectrophotometric analysis. DNA and protein sequences were obtained from genome database (http://genome.jgi.doe.gov/vista_embed/?organism = Mucci2). Specific primers to amplify target genes construct disruption vectors and probes are indicated in Table 4. Cloning experiments were performed using pBluescript and *Escherichia coli* strain DH5α [46].

### Target gene knockout and fungal transformation

PCR reactions to construct deletion vectors were performed with Herculase II Fusion DNA Polymerase (Agilent Technologies, Santa Clara, CA) using specific primers listed in Table 4. Briefly, the 5′ and 3′ flanking sequences of the target gene were obtained by PCR amplification of CBS277.49 gDNA using specific primers with 5′ tails (30 bp) complementary to pyrG, and the resulting 1 Kb fragments were fused to the selective marker pyrG gene. The whole replacement fragment was PCR amplified using primers of the flanking regions and cloned into the vector pBluescript by blunt end ligation at *Sma* I site (Fig. 4A).

Transformation was carried out on protoplasts as previously described [47] and the homologous recombination events were confirmed by Southern analysis of gDNA using the indicated probes and restriction enzymes (Fig. 4B) as previously described [48] using the non-isotopic digoxigenin labeling kit (Roche Applied Science).

### Statistical analysis

The Mantel-Cox method was used to assess statistical significance of differences in survival among groups. Fungal burden data were expressed as mean ± standard deviation, and differences among organs were tested by two-tailed t-test. Data were analyzed using Graph Pad Prism software and the values *P* < 0.05 were considered statistically significant.

## Supplementary Material

143529_supp.zip
