# Supplementary material for: Understanding Mucor circinelloides pathogenesis by comparative genomics and phenotypical studies
Source: Virulence. 2018 Apr 18;9(1):707–20. doi: 10.1080/21505594.2018.1435249 (PMC5955452; doi:10.1080/21505594.2018.1435249)
Supplement: 143529_supp.zip [file kvir-09-01-1435249-s001.zip › 143529_supp/2017VIRULENCE0146R2-f06-z-4c.pptx]

## Slide 1
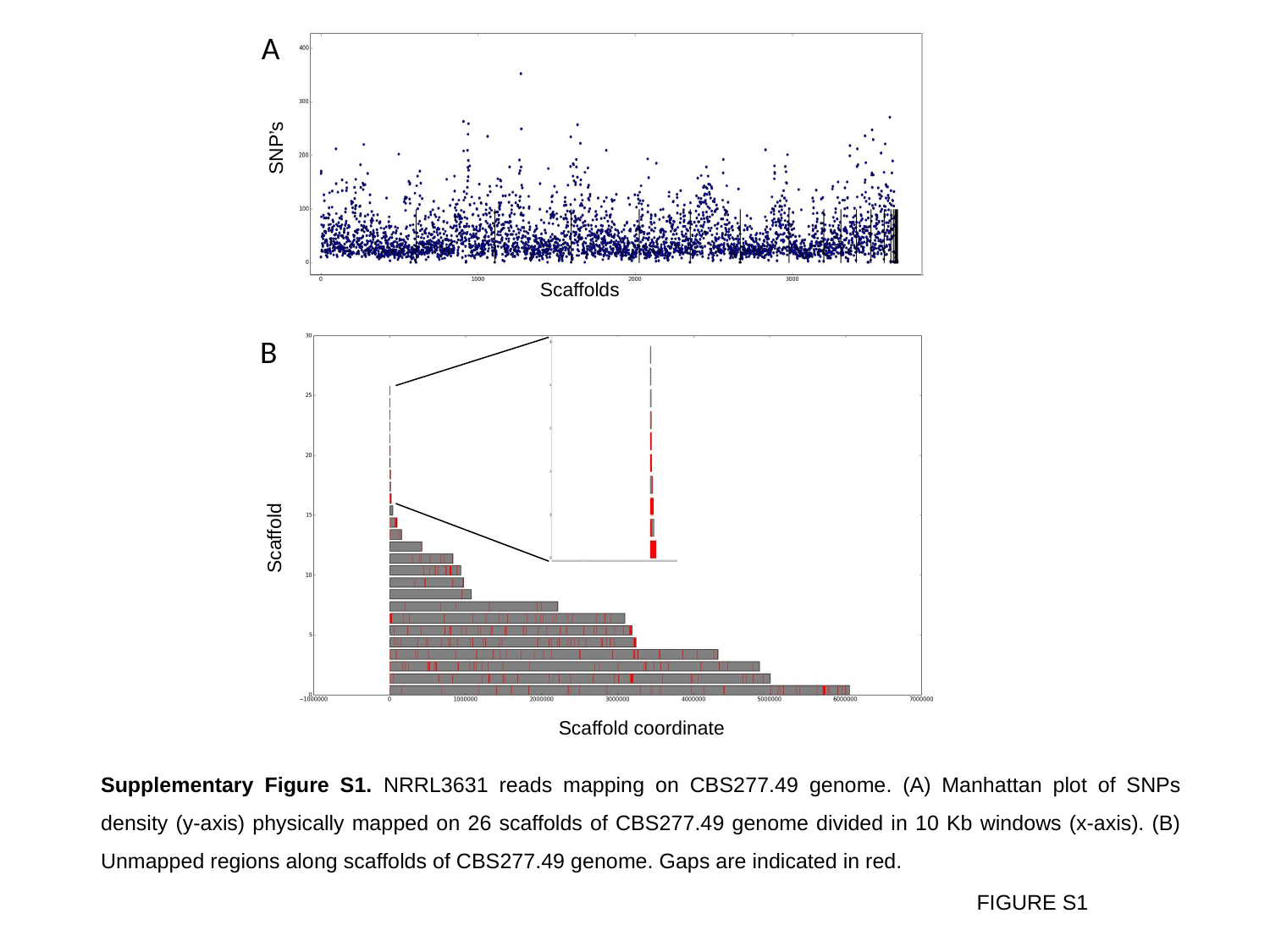

A
SNP’s
Scaffolds
B
Scaffold
Scaffold coordinate
Supplementary Figure S1. NRRL3631 reads mapping on CBS277.49 genome. (A) Manhattan plot of SNPs density (y-axis) physically mapped on 26 scaffolds of CBS277.49 genome divided in 10 Kb windows (x-axis). (B) Unmapped regions along scaffolds of CBS277.49 genome. Gaps are indicated in red.
FIGURE S1
