# Supplementary material for: Understanding Mucor circinelloides pathogenesis by comparative genomics and phenotypical studies
Source: Virulence. 2018 Apr 18;9(1):707–20. doi: 10.1080/21505594.2018.1435249 (PMC5955452; doi:10.1080/21505594.2018.1435249)
Supplement: 143529_supp.zip [file kvir-09-01-1435249-s001.zip › 143529_supp/2017VIRULENCE0146R2-f07-z-4c.pptx]

## Slide 1
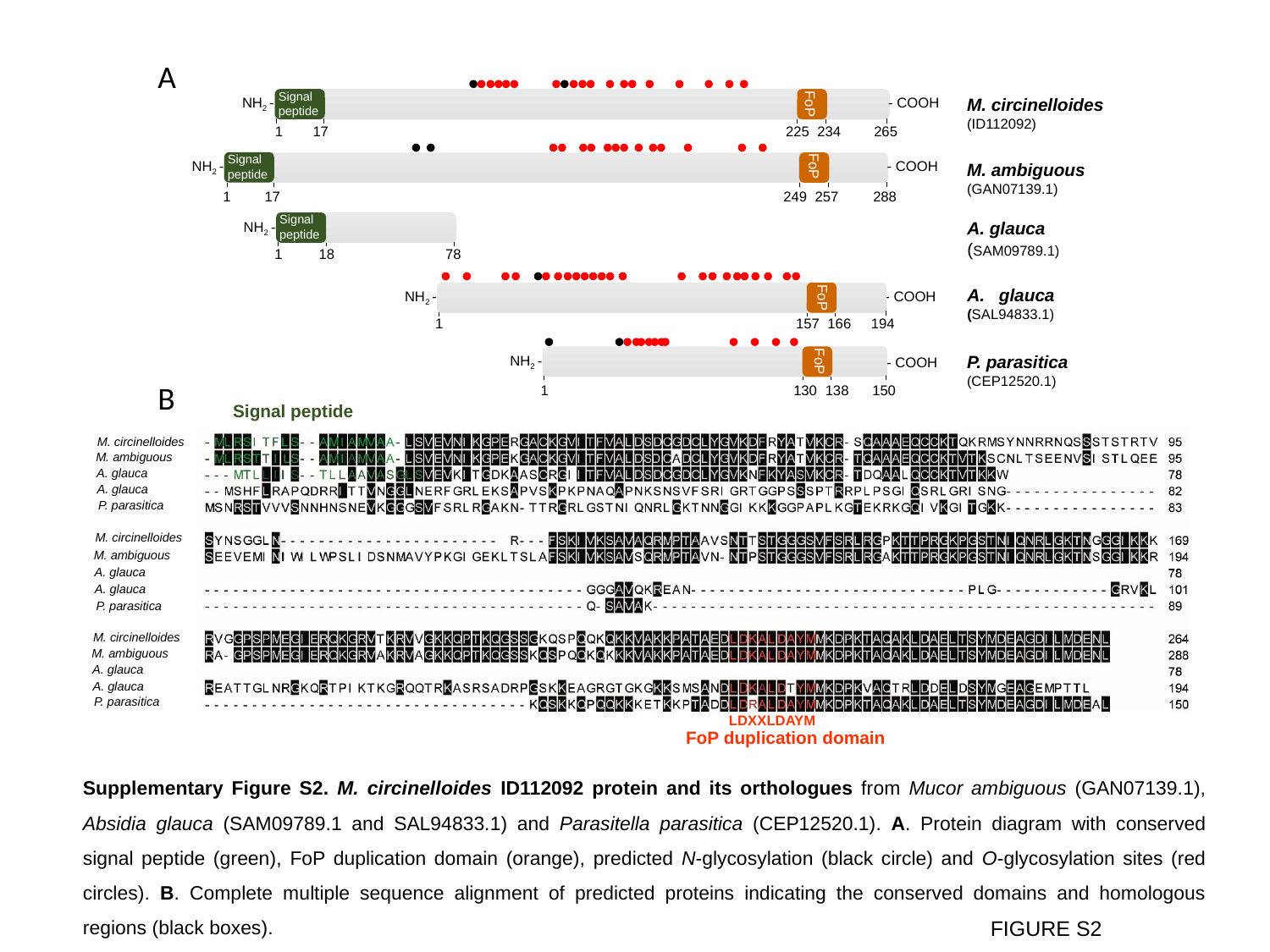

A
Signal peptide
M. circinelloides
(ID112092)
- COOH
NH2 -
FoP
1
17
225
234
265
Signal peptide
NH2 -
- COOH
FoP
M. ambiguous
(GAN07139.1)
1
17
249
257
288
Signal peptide
A. glauca (SAM09789.1)
NH2 -
1
18
78
glauca
(SAL94833.1)
NH2 -
- COOH
FoP
1
157
166
194
P. parasitica
(CEP12520.1)
NH2 -
FoP
- COOH
1
130
138
150
B
Signal peptide
M. circinelloides
M. ambiguous
A. glauca
A. glauca
P. parasitica
M. circinelloides
M. ambiguous
A. glauca
A. glauca
P. parasitica
M. circinelloides
M. ambiguous
A. glauca
A. glauca
P. parasitica
LDXXLDAYM
FoP duplication domain
Supplementary Figure S2. M. circinelloides ID112092 protein and its orthologues from Mucor ambiguous (GAN07139.1), Absidia glauca (SAM09789.1 and SAL94833.1) and Parasitella parasitica (CEP12520.1). A. Protein diagram with conserved signal peptide (green), FoP duplication domain (orange), predicted N-glycosylation (black circle) and O-glycosylation sites (red circles). B. Complete multiple sequence alignment of predicted proteins indicating the conserved domains and homologous regions (black boxes).
FIGURE S2
