# Supplementary material for: Understanding Mucor circinelloides pathogenesis by comparative genomics and phenotypical studies
Source: Virulence. 2018 Apr 18;9(1):707–20. doi: 10.1080/21505594.2018.1435249 (PMC5955452; doi:10.1080/21505594.2018.1435249)
Supplement: 143529_supp.zip [file kvir-09-01-1435249-s001.zip › 143529_supp/2017VIRULENCE0146R2-f08-z-4c.pptx]

## Slide 1
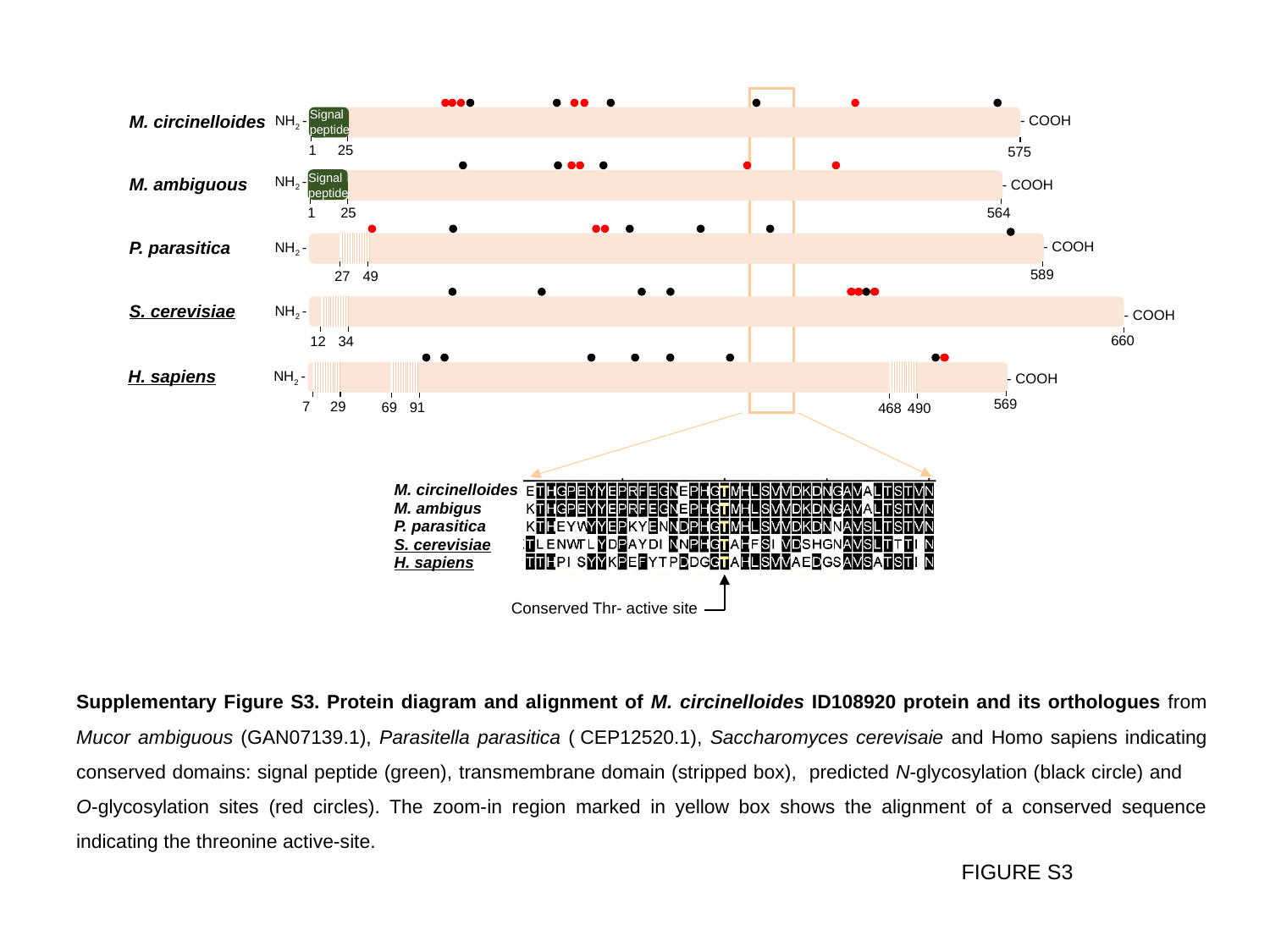

Signal peptide
M. circinelloides
- COOH
NH2 -
1
25
575
Signal peptide
NH2 -
M. ambiguous
- COOH
1
25
564
P. parasitica
- COOH
NH2 -
589
27
49
S. cerevisiae
NH2 -
- COOH
660
12
34
H. sapiens
NH2 -
- COOH
569
7
29
69
91
468
490
M. circinelloides
M. ambigus
P. parasitica
S. cerevisiae
H. sapiens
Conserved Thr- active site
Supplementary Figure S3. Protein diagram and alignment of M. circinelloides ID108920 protein and its orthologues from Mucor ambiguous (GAN07139.1), Parasitella parasitica ( CEP12520.1), Saccharomyces cerevisaie and Homo sapiens indicating conserved domains: signal peptide (green), transmembrane domain (stripped box), predicted N-glycosylation (black circle) and O-glycosylation sites (red circles). The zoom-in region marked in yellow box shows the alignment of a conserved sequence indicating the threonine active-site.
FIGURE S3
