# Supplementary material for: Understanding Mucor circinelloides pathogenesis by comparative genomics and phenotypical studies
Source: Virulence. 2018 Apr 18;9(1):707–20. doi: 10.1080/21505594.2018.1435249 (PMC5955452; doi:10.1080/21505594.2018.1435249)
Supplement: 143529_supp.zip [file kvir-09-01-1435249-s001.zip › 143529_supp/2017VIRULENCE0146R2-f09-z-4c.pptx]

## Slide 1
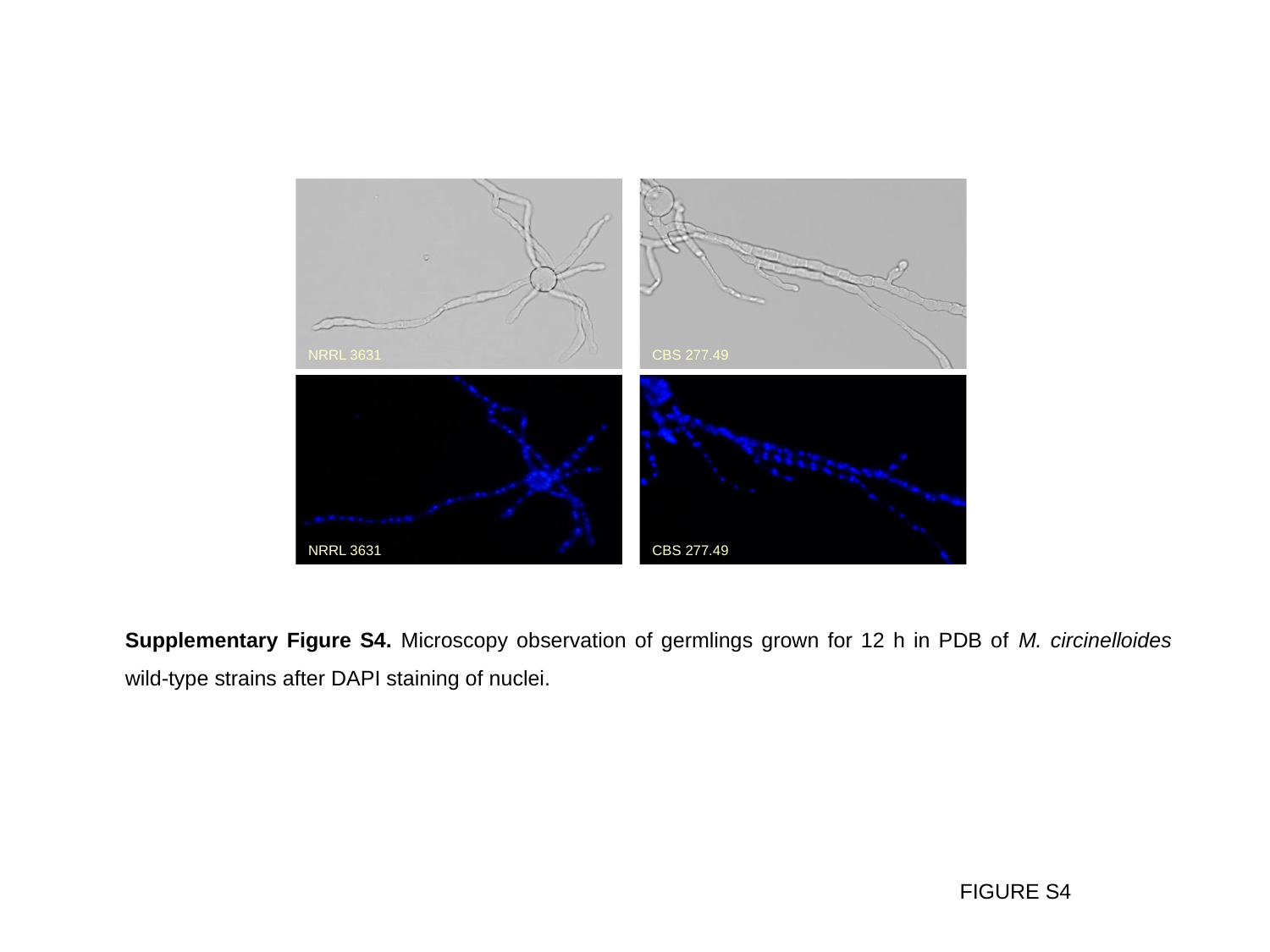

NRRL 3631
CBS 277.49
NRRL 3631
CBS 277.49
Supplementary Figure S4. Microscopy observation of germlings grown for 12 h in PDB of M. circinelloides wild-type strains after DAPI staining of nuclei.
FIGURE S4
