# Supplementary material for: Understanding Mucor circinelloides pathogenesis by comparative genomics and phenotypical studies
Source: Virulence. 2018 Apr 18;9(1):707–20. doi: 10.1080/21505594.2018.1435249 (PMC5955452; doi:10.1080/21505594.2018.1435249)
Supplement: 143529_supp.zip [file kvir-09-01-1435249-s001.zip › 143529_supp/2017VIRULENCE0146R2-file002.docx]

|  | CBS 277.49 | |
| --- | --- | --- |
|  | *Lenght (µm)* | *Nuclei number* |
| Hypha 1 | 129.61 | 23 |
| Hypha 2 | 131.74 | 21 |
| Hypha 3 | 259.43 | 47 |
| Hypha 4 | 43.47 | 24 |
| Hypha 5 | 53.32 | 10 |
| Hypha 6 | 77.28 | 13 |
| Hypha 7 | 134.59 | 35 |
| 20.9 nuclei / 100 µm hypha | | |
|  |  | |
|  | NRRL 3631 | |
|  | *Lenght (µm)* | *Nuclei number* |
| Hypha 1 | 318.0 | 67 |
| Hypha 2 | 206.8 | 32 |
| Hypha 3 | 115.23 | 25 |
| Hypha 4 | 276.85 | 46 |
| Hypha 5 | 180.1 | 33 |
| Hypha 6 | 67.89 | 19 |
| 19.1 nuclei / 100 µm hypha | | |

**Supplementary Table S1**. Nuclei number determined by Dapi staining and microscopic observation in *M. circinelloides* strains CBS 277.49 and NRRL 3631.
